# Elucidating the mechanism of the considerable mechanical stiffening of DNA induced by the couple Zn^2+^/Calix[4]arene-1,3-O-diphosphorous acid

**DOI:** 10.1038/s41598-018-19712-4

**Published:** 2018-01-19

**Authors:** Yannick Tauran, Mehmet C. Tarhan, Laurent Mollet, Jean Baptiste Gerves, Momoko Kumemura, Laurent Jalabert, Nicolas Lafitte, Ikjoo Byun, Beomjoon Kim, Hiroyuki Fujita, Dominique Collard, Florent Perret, Mickael Desbrosses, Didier Leonard, Christelle Goutaudier, Anthony W. Coleman

**Affiliations:** 1 LMI CNRS UMR 5615, Université Lyon 1, Villeurbanne, 69622 France; 2 LIMMS/CNRS-IIS UMI 2820, Institute of Industrial Science, The University of Tokyo, Tokyo, 153-8505 Japan; 3 CNRS/IIS/COL/Lille 1 SMMiL-E project, 59046 Lille Cedex, France; 4 CIRMM, Institute of Industrial Science, The University of Tokyo, Tokyo, 153-8505 Japan; 5 Univ. Lille, CNRS, Centrale Lille, ISEN, Univ. Valenciennes, UMR 8520 - IEMN, Lille, F59000 France; 6 Univ. Lyon, Université Claude Bernard Lyon 1, CNRS, CPE Lyon, ICBMS UMR 5246, 43 Boulevard du 11 Novembre 1918, F-69622 Lyon, France; 7 ISA, UMR 5280, Univ. Lyon 1, Villeurbanne, F69100 France

## Abstract

The couple Calix[4]arene-1,3-O-diphosphorous acid (C4diP) and zinc ions (Zn^2+^) acts as a synergistic DNA binder. Silicon NanoTweezer (SNT) measurements show an increase in the mechanical stiffness of DNA bundles by a factor of >150, at Zn^2+^ to C4diP ratios above 8, as compared to Zinc alone whereas C4diP alone decreases the stiffness of DNA. Electroanalytical measurements using 3D printed devices demonstrate a progression of events in the assembly of C4diP on DNA promoted by zinc ions. A mechanism at the molecular level can be deduced in which C4diP initially coordinates to DNA by phosphate-phosphate hydrogen bonds or in the presence of Zn^2+^ by Zn^2+^ bridging coordination of the phosphate groups. Then, at high ratios of Zn^2+^ to C4diP, interdigitated dimerization of C4diP is followed by cross coordination of DNA strands through Zn^2+^/C4diP inter-strand interaction. The sum of these interactions leads to strong stiffening of the DNA bundles and increased inter-strand binding.

## Introduction

DNA is one of the key bio-polymers in life, encoding the information required to build organisms. Defects in DNA can occur either via natural mutations or through external damage^1^. Such defects are the cause of a vast range of diseases, from Huntington’s disease to cancer, although some mutations may be beneficial^2^.

In recent years, the rise of artificial nanostructures based on DNA often coupled to metal ions, has been pioneered by Seeman^3^. Artificial DNA binders present a great interest in the biomedical field^4^. They can be used in various ways, such as the transportation of DNA through the cell membrane in transfection or gene therapy^5^ for blocking a specific DNA sequence to avoid the transcription of a specific gene^6^ or arresting DNA replication in cancer treatment^7^.

The calix[n]arenes, are well known for their ability to interact with a wide range of biomolecules including amino acids, nucleotides, surfactants, proteins and DNA^8^, leading to a wide panoply of biological applications^9^. Cationic calix[n]arene derivatives have been reported to be efficient DNA binders^10^. However, such derivatives present several drawbacks in biology, including toxicity due to the destabilization of cellular membranes possessing negative potential or non-specific electrostatic aggregation by negatively charged DNA^11^. Protein ligands of DNA (finger zinc proteins, transcription factors, nucleases) interact mainly through conjoint coordination of metal cationic ions^12^. Here, we investigate the couple of an anionic calix[n]arene and a metal cation for DNA binding.

**Figure 1:**
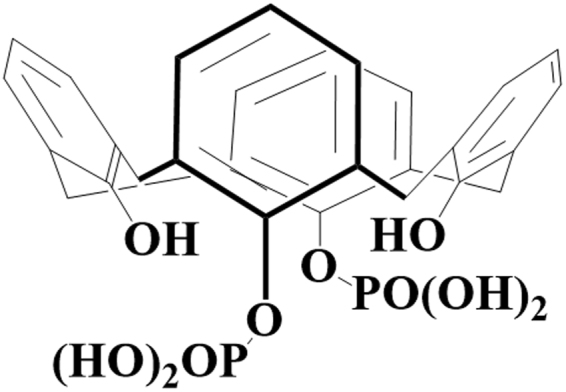
Chemical structure of calix[4]arene-1,3-O-diphosphorous acid (C4diP calix[4]arene).

Among the anionic calix[n]arenes, calix[4]arene-1,3-O-diphosphorous acid (C4diP; Fig. 1) has attracted interest in view of low hematoxicity, reasonable aqueous solubility and anti-cancer properties^13^. The anti-cancer effects are necrotic and not the apoptotic effects normally associated with anti-cancer drugs. C4diP possesses a wide therapeutic window, with toxicity much higher for cancer proliferative cells as compared to normal proliferative cells. The anti-cancer properties of C4diP are unique for the calix[n]arenes as neither the *para*-sulphonato nor the O-alkyl carboxylate derivatives show any activity against cancer cell lines^13^. The use of Electrospray/Mass Spectrometry (ES/MS) demonstrated that C4diP was able to complex amino acids and metal ions with a remarkable selectivity and high affinity for histidine and zinc ion, Zn^2+^, in a triple complex^14^.

Zn^2+^ is an essential metal ion, known for its versatility of interaction with DNA either via nucleobases or via phosphate groups when coordinated with macrocyclic polyamines^15^. Zn^2+^ has a high concentration in human cells (200–300 µM) and shows unique coordination properties with DNA^16^.

In view of the above the couple Zn^2+^/C4diP would seem to be a good candidate for binding to DNA. However, the investigation of such a ternary complex by conventional physical methods, is limited in terms of sensitivity^14^, or in terms of what physical information (for example mechanical and electrical effects) can be accessed.

In the current paper, two novel technologies: 3D printing with post-fabrication chemical modification, and Silicon NanoTweezers (SNTs) for measurement at the molecular level of mechanical properties, have been used to elucidate the assembly mechanism of the couple Zn^2+^/C4diP as a synergistic binding system for DNA.

**Figure 2:**
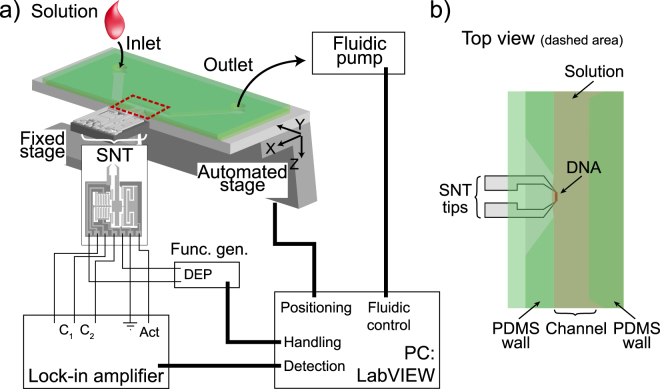
(**a**) The SNT device setup. (**b**) Immersion of the SNT tips in the channel of the microfluidic device, allowing the captured DNA bundle to interact with different solutions without passing through the meniscus, and hence avoiding strong mechanical stress on the bound DNA bundles.

The combination of SNT with a suitable microfluidic device provides a new methodology for measuring the mechanical properties of biopolymers^17^ including the changes selectively induced by magnesium ions. Briefly, the method consists of a microelectromechanical systems (MEMS) device possessing two opposed tips that can trap biological fibres, e.g. bundles of DNA, using dielectrophoresis-assisted combing^17^. One of the opposed tips is stationary and the other one is mechanically connected (while being isolated electrically) to an actuator and a displacement sensor (Fig. 2a). Outputs of the sensor (C1 and C2) are fed into a lock-in amplifier, which drives the actuator. According to the displacement sensor readings, the lock-in-amplifier changes the frequency of the signal to keep the system running at a constant phase. This phase-lock-loop enables real-time determination of the resonance frequency and the amplitude at that frequency of the DNA trapped between the tweezers tips. The tips are inserted into the cavity of the microfluidic device, which is connected to a microfluidic canal allowing the solution around the SNTs to be exchanged (Fig. 2b). Thus it is possible to titrate ligands and to observe the mechanical effect of the ligand as a function of their concentration, we have recently demonstrated the use of the method for observation of how physiological cations affect the mechanical properties of DNA and in particular a small but significant effect for Mg^2+^ at the intra-nuclear concentration^18^. Through the change of the resonance frequency and amplitude of DNA resonance signal induced by the ligand, we can deduce the change of stiffness and viscous losses, in real time and hence study mechanical properties of the biopolymer as it responds to ligands.

## Results

**Figure 3:**
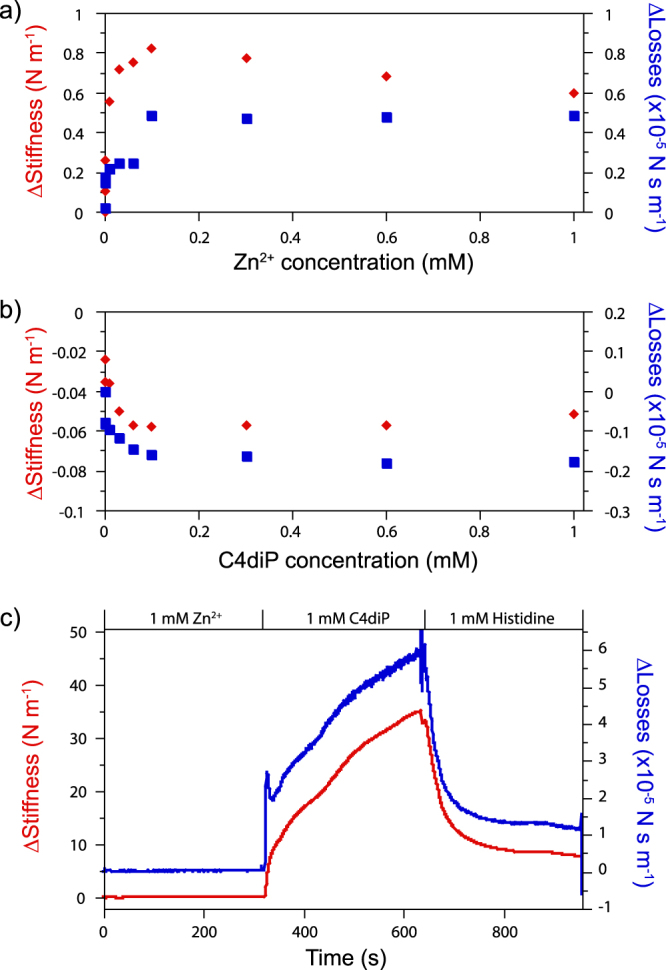
Mechanical measurement of DNA stiffness (red diamonds, left scale) and DNA viscous losses (blue squares, right scale) as a function of (**a**) Zn^2+^ and (**b**) C4diP concentration. Plate (**c**) shows real time measurements of DNA stiffness (red) and viscous losses (blue) after consecutive injections of Zn^2+^, C4diP and Histidine (Solution changes are shown between arrows). The real change for Zn^2+^ alone is shown in the inset.

The mechanical effect of Zn^2+^ and C4diP on DNA bundles were measured separately (Fig. 3a and b). It must be emphasised that bundle size can vary quite strongly between bundles making direct numerical comparison between bundles impossible. For Zn^2+^, DNA appears to exhibit two regimes of linear increase in stiffness and viscous losses with a break point at 0.1 mM. For C4diP, a smaller exponential decline in stiffness and viscous losses is observed (Supplementary Figs 1–8). In a separate experiment now using only one bundle, when C4diP has been injected after Zn^2+^, a dramatic increase of DNA stiffness to 33.5 N m^−1^ and DNA viscous losses of 5.65 × 10^−5^ N s m^−1^ has been observed (Fig. 3c, the inset shows the effect of Zn^2+^ on this bundle, <0.2 N m^−1^. The couple Zn^2+^/C4diP increases by a factor of >150 the stiffness of DNA compared to Zn^2+^ at the same concentration. Injection of histidine reverses the effect, (the final decrease to the starting level is extremely slow). This complexation is in agreement with the observation, using ES/MS, of a triple complex C4diP/Zn^2+^/Histidine complex by Perret^14^. Other experiments showed much larger increases in the stiffness but were often accompanied by a lack of return to the base line by Histidine. This is not unexpected as the K_ass_ for the triple complex may not be sufficiently high to ensure complete removal of the Zn^2+^/C4diP couple.

To better understand this dramatic increase on stiffness, we have studied the mechanical effect of DNA as a function of a Zn^2+^/C4diP ratio (with a constant concentration of C4diP at 0.2 mM), using the microfluidic device coupled to the SNTs.

**Figure 4:**
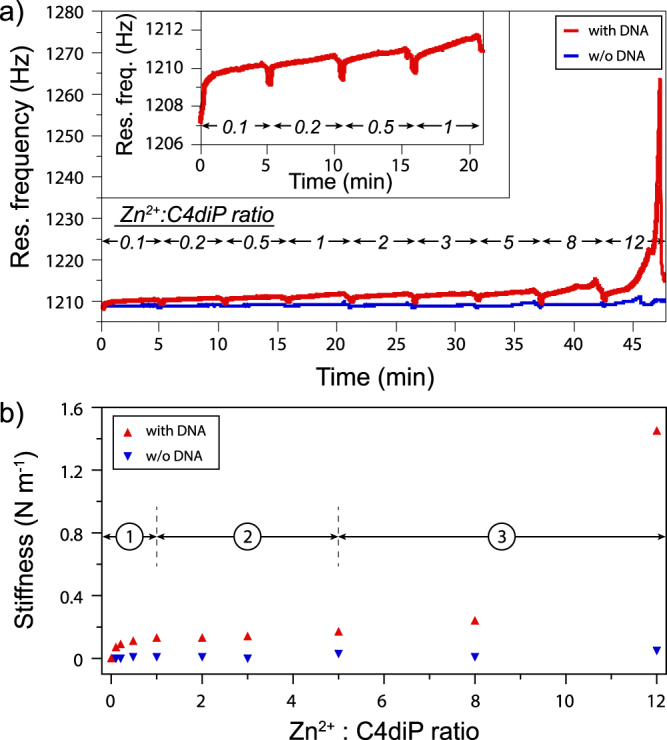
(**a**) Real-time measurements of the resonance frequency after consecutive injections of Zn^2+^/C4diP solutions at different ratios, measurement times 5 minutes each. (**b**) The average of stiffness from Fig. 2a during the last 40 seconds of the injection, at each injected Zn^2+^/C4diP ratio. In blue is shown the stiffness response of SNTs in the absence of DNA.

In Fig. 4, three different steps can be seen. Initially, the stiffness and the viscous losses (Supplementary Figs 9 and 10) of DNA increase up to a ratio 1 for Zn^2+^ vs C4diP. Then for ratios from 1 to 5, a plateau is observed where the mechanical parameters are approximately stable. At a ratio superior to 5, the stiffness and viscous losses increase considerably, with a very large increase at a Zn^2+^: C4diP ratio of 12. To aid understanding both curves should be considered.

**Figure 5:**
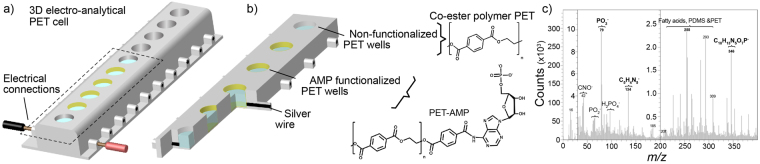
(**a**) Schematic representation of 3D printed PET electro-analytical cells for resistivity measurements. (**b**) The wells are filled with a solution (blue) to measure resistivity for AMP functionalized wells (orange/yellow) and non-treated wells (grey). Lateral inlets are filled with silver to measure the resistivity at the interface polymer/solution by multimeter probes (in black and red). (**c**) The ToF-SIMS spectra after surface functionalization.

In an inter-method approach, a 3D printed electro-analytical cell was constructed (Fig. 5), to allow better understanding of how C4diP, Zn^2+^ or the couple Zn^2+^/C4diP can assemble on a nucleotide functionalized surface. A series of 10 resistivity cells comprising the device was designed using Inventor Professional (Autodesk Education Community, France) and then printed using Fused Filament Deposition by means of a Leapfrog Creatr 3d Printer (Leapfrog, UK). The 3D electro-analytical cell itself was made of Polyethylene Terephthalate (PET) polymer. Eight modified cells were used to allow elimination of statistical out-riders (about 20%), whilst two cells are pure water references.

After printing, the lateral channels of the electro-analytical cells were filled with a conductive silver paste (Sigma Aldrich). Then, the multi-cell device was heated for 2 hours at 120 °C in an oven to anneal the silver paste. The 3D cells were surface functionalized, by ester aminolysis^19^, using Adenosine Monophosphate (AMP; 0.2 mL at 0.1 mM) and the reaction was carried out during 24 hours, under a glass cover, these conditions had been previously determined as optimal. Here the primary amine of the nucleic acid, AMP, reacts with the ester group of the PET surface to generate amide bonds. For the purpose of optimisation ten flat surfaces were printed and the modification of the surface was monitored using contact angle measurements. The initial value was 80.8° and declined to a constant value of 68.6° after 24 hours. The presence of bound AMP groups on the PET was conclusively determined using ToF/SIMS (Supplementary Fig. 11).

The choice of AMP is arbitrary as if the binding occurs to the phosphoester backbone, the nature of the nucleic base will have no effect. Indeed this is the case, as essentially identical results were observed using GMP to functionalise the surface. Then, the 3D device was washed with de-ionized water and dried in the absence of dust.

**Figure 6:**
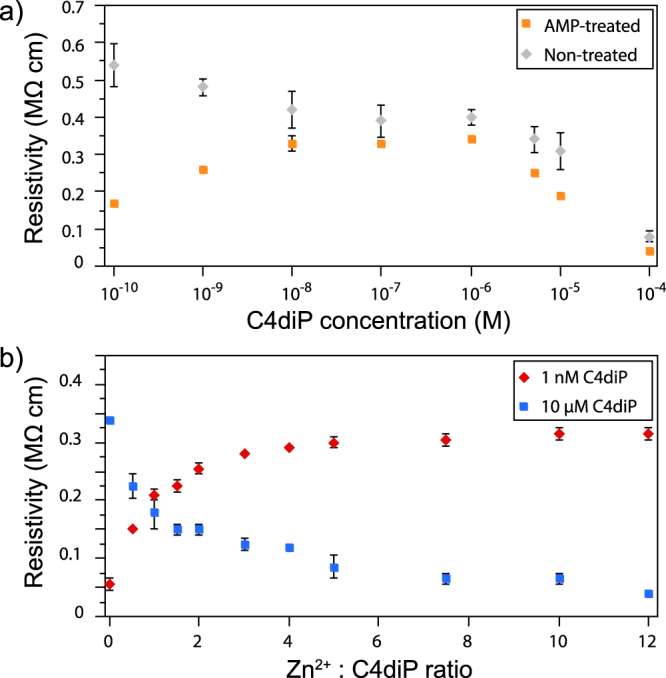
(**a**) Resistivity of a concentration series of C4diP on AMP treated (orange) and non-treated surfaces (grey). (**b**) Resistivity of Zn^2+^/C4diP solutions at different ratios of Zn^2+^/C4diP, with C4diP concentration at 1 nM (red) and 10 µM (blue). Experiments were performed in duplicate.

The resistivity measurements were carried out by connection of the probes of a numerical multimeter (MX54C, Metrix, France) to the silver filled lateral channels (Supplementary Fig. 12). As expected Zn^2+^ solutions show a decreasing resistivity as a matter of concentration on both non-treated and treated surface. Unlike Zn^2+^, C4diP shows a different behaviour when placed in AMP functionalized wells (Fig. 6a). Three effects occur. Firstly, the resistivity increases between 1 × 10^−10^ M to 1 × 10^−8^ M in C4diP concentration. Secondly, a plateau is reached between for C4diP concentrations of 1 × 10^−8^ M to 1 × 10^−6^ M. Thirdly, the resistivity decreases at C4diP concentrations superior to 1 × 10^−6^ M.

The unexpected increase of resistivity at the first step can be explained by the assembly of C4diP on the surface such that the aromatic face of C4diP is oriented away from the surface. At higher concentration, C4diP could arrange in an interdigitated bilayer structure reported by Suwinska^20^, where aromatic faces, π-π interact to give a capsule and exhibit a charged phosphonate group at the surface. Subsequently, the complex Zn^2+^/C4diP has been studied at two concentrations of C4diP on an AMP functionalized surface (Fig. 6b). This shows that at 1 nM (red curve) when the ratio Zn^2+^/C4diP increases, resistivity increases, implying the Zn^2+^ cations coordinate between the C4diP and AMP phoshonate groups. The apolar cavity of the macrocycle is thus oriented away from the surface. At the C4diP concentration of 10 µM (blue curve) the C4diP molecules interdigitate and Zn^2+^ promotes the orientation of the capsules with the negatively charged phosphonate group oriented away from the polymer surface.

## Discussion

**Figure 7:**
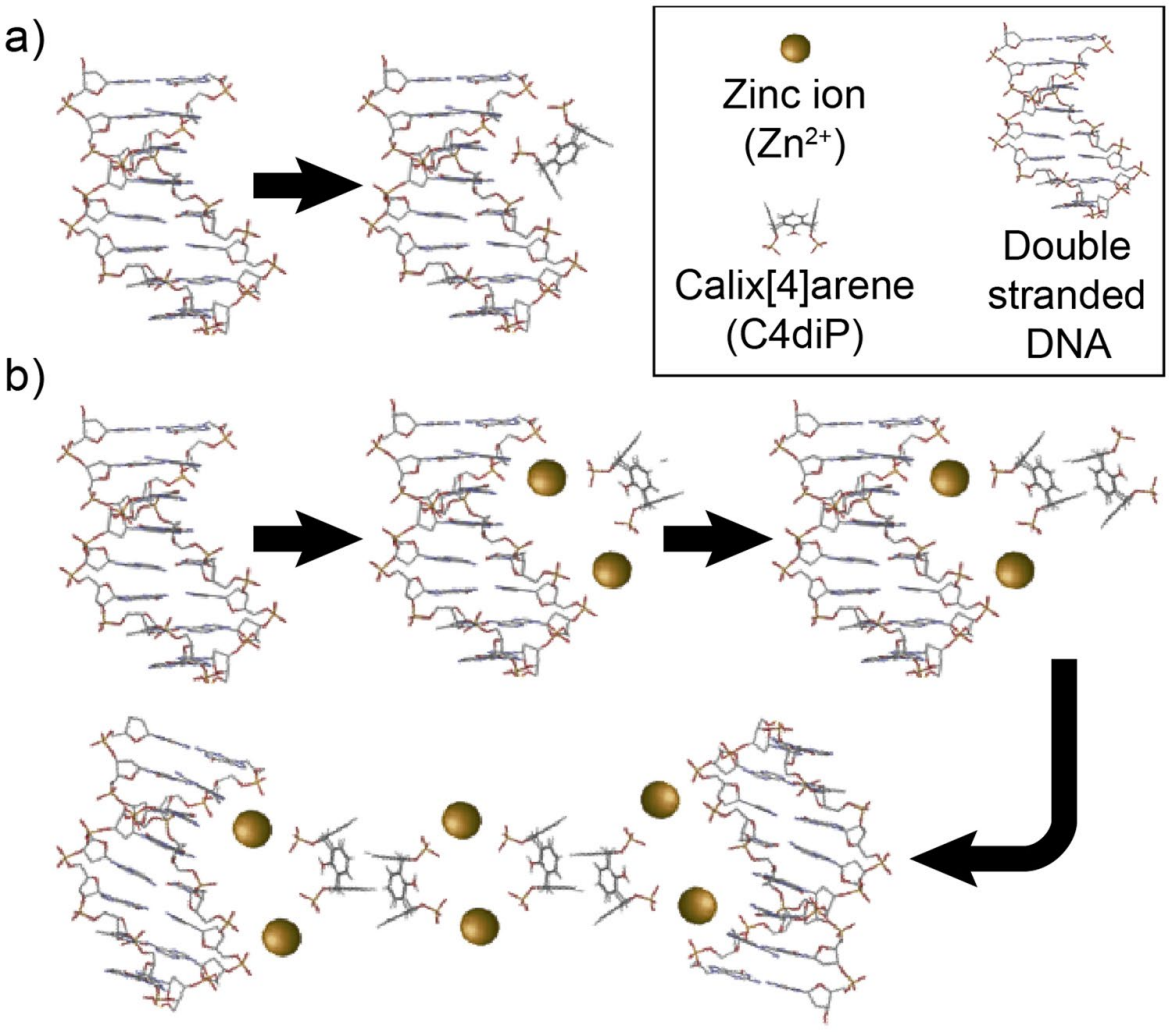
Proposed structures of different assemblies of the calix[n]arene C4diP, Zn^2+^ and DNA complex. The nature of the assembly is dependent on the Zn^2+^/C4diP ratio and their concentrations. (**a**) C4diP, H-bonded, assembly on DNA phosphate groups at low C4diP concentration (**b**) Zn^2+^ stabilized C4diP binding at low C4diP concentration. At higher concentration, C4diP acts as an interdigitated capsule and stabilised by Zn^2+^ coordination to DNA. Finally, DNA aggregates at high C4diP concentration and high Zn^2+^/C4diP ratio.

A molecular mechanism dependant on the concentration and ratio of the complex Zn^2+^/C4diP can be deduced explaining the considerable changes in the mechanical properties of DNA. At low C4diP concentrations, the molecule could H-bond to the phospho-ester chain. However, we rule out this possibility as it cannot explain how Zn^2+^ induced inter-strand interactions could occur. Such behaviour is well known for divalent cation bridging of polysaccharides which is generally accompanied by gelling and thus modification of the mechanical properties^21^. In the presence of Zn^2+^, the cation as expected coordinates, in abridging manner, between C4diP and DNA and thus increases stability. Thus, C4diP is linked along the DNA with the apolar aromatic face projecting out, generating an increase in the resistivity (Fig. 7a). At higher C4diP concentration and low ratio of Zn^2+^/C4diP, the calix[n]arene will self-assemble in the classic interdigitated dimeric structure^22^, projecting the phosphate groups outward, in agreement with a decrease in resistivity and no significant mechanical change (Fig. 7b). Finally at higher C4diP concentration and a high ratio of Zn^2+^/C4diP, superior to 5, DNA aggregation occurs where zinc and C4diP dimers bridge DNA strands, leading to considerable stiffening and increase in inter-strand friction (Fig. 6b).

In order to verify the aggregation behaviour, Dynamic Light Scattering experiments were undertaken, all measurements were taken 30 minutes after mixing. These show, the presence of objects of diameter 43 nm and 142 nm for DNA alone, in the presence of Zn^2+^ cations the size increases to 44 nm and 303 nm, for DNA in the presence of C4diP the sizes are now 51 nm and 274 nm. In the presence of C4diP and Zn^2+^ cations at a ration of 1:10 precipitation occurs, due aggregation, and no objects can be observed (Supplementary Fig. 13).

In conclusion, the calix[n]arene C4diP can use Zinc ion coordination to interact with DNA. A molecular mechanism in three steps has been also proposed where the assembly of the complex along the DNA is driven by the ratio Zn^2+^/C4diP. Finally, a remarkable mechanical effect inducing DNA condensation has been shown from a specific ratio (ratio Zn^2+^/C4diP ≥ 5). The study has required the inter-methodology use of novel technologies: SNTs and 3D printing.

## Methods

### Synthesis and characterization of Calix[4]arene-1,3-O-diphosphorous acid (C4diP)

All chemicals were purchased from ACROS Organics or Sigma Aldrich and used without further purification. Solvents were of chemical grade and were used without any purification.

Calix[4]arene-1,3-O-diphosphorous acid (C4diP) as per the method of Markovsky and Kalchenko^22^. All the physical characteristics of the synthetized calix[n]arene correspond to the literature values.

### ToF-SIMS Measurements

ToF-SIMS measurements were carried out using a Physical Electronics (Chanhassen, USA) TRIFT III instrument operated with a pulsed 22 keV ^197^Au^+^ ion gun (ion current of 2 nA) rastered over a 300 μm × 300 μm area, resulting in an ion dose of 2.9 × 10^9^ ions cm^−2^ per analysis. An electron gun was operated in pulsed mode at low electron energy for charge compensation. Ion dose was kept below the static conditions limit. Data were analyzed using WinCadence^TM^ software, version 5.3.0.12. Mass calibration was performed using H^−^, CN^−^, C_2_HO^−^, CNO^−^ and C_3_H_3_O_2_^−^.

### Real-time measurements with SNT

The details of real-time measurements with SNT were reported earlier^17^. On an upright microscope stage (Keyence VHX-500), SNT was kept stationary at all times while the microfluidic device were positioned with high accuracy using a computer controlled motorized 3D stage (SLC-1720S-STU-XYZ, SmarAct GmbH). SNT was driven with a lock-in-amplifier (Signal Recovery AMETEK 7270 DSP), which was locked on the phase of the SNT via the capacitive displacement sensor readings. First, a DNA bundle was captured from a DNA solution (0.175 mg ml^−1^ λ-phage DNA) by dielectrophoresis-assisted lateral combing. Then, SNT tips (with the captured DNA bundle) were inserted in the channel via a side opening of the microfluidic device. Connected to a vacuum pump (AF1 Dual, Elveflow), the microfluidic device allowed changing the solution in the channel. Therefore, effects of different solutions could be tested on the same DNA bundle.

In the experiments, we examined the effect of Zn^2+^ and C4diP solutions by testing them separately (increasing concentrations from 100 nM to 1 mM for C4diP and 100 nM to 3 mM for Zn^2+^), consecutively (1 mM Zn^2+^ followed by 1 mM C4diP with a final injection of 1 mM Histidine) and simultaneously (at Zn^2+^:C4 diP ratios varying from 0.5 to 12). Experiments started after inserting the captured DNA bundle in the liquid. For each solution, 5 min of incubation time was followed by a 20-s flow to exchange the solution using the vacuum pump.

A LabVIEW program controlled all equipment and recorded the resonance frequency, amplitude (at resonance) and thus the Q-factor values throughout the experiments. Those values were then used to calculate the mechanical properties of the DNA bundle *i.e*. stiffness (*k*_*mb*_) and viscous losses (*η*_*mb*_), using equations (1) and (2):

1 $${f}_{R}(t)=\frac{1}{2\pi }\sqrt{\frac{k+{k}_{mb}(t)}{M}},$$

2 $$Q(t)=\frac{\sqrt{(k+{k}_{mb}(t)).M}}{\eta +{\eta }_{mb}(t)}$$

### Electrochemical Cell 3D Printing and measurements

The lateral channels of the 3D electroanalytical devices were metallized and made conductive by filling with silver conductive paste (Sigma Aldrich). Then, the device has been heated for 2 hours at 120 °C in an oven, in order to anneal the silver paste.

For the surface functionalization treatment, 1 mL of a saturated solution Adenosine Mono Phosphate AMP (Sigma-Aldrich) was deposited in the wells of the 3D device, covered with a glass lid and the reaction was carried out, at room temperature, during 24 hours. Finally, the device was washed with DI water and dried under a cover. For the non-functionalized surface, 1 mL of DI Water was used instead of the AMP solution.

Functionalization has been characterized by ToF-SIMS (Supplementary Fig. 7). A signal at m/z = 134 corresponds to the presence of AMP nucleotide base. When the 3D object has been functionalized with Guanosine Mono Phosphate GMP, a peak at m/z = 348 corresponding to GMP minus one OH function is detected (data not shown).

Solutions in a concentration range from 0.1 nM to 1 µM of C4diP and Zinc nitrate (Supplementary Fig. 8) were prepared (Sigma Aldrich). Ratios of Zn^2+^/C4diP varying from 0.1 to 12 at a constant concentration of C4diP at 1 nM and 10 µM have been also prepared.

Then, the resistivity of each solution has been monitored by connecting the probes of a digital multimeter (MX54C, Metrix) to the metallized lateral channels of the 3D device.

### Dynamic Light Scattering

The mean particle size (diameter, nm) and the polydispersity index (PdI) of the objects were measured by dynamic light scattering using a NanoZS instrument (Malvern Instruments UK), which analyses the fluctuations of scattered light intensity generated by diffusion of the particles in a diluted suspension. The measurements were carried out at 25 °C. Experiments were performed in triplicate.

## Electronic supplementary material

Supplementary Information
