# Supplementary material for: Elucidating the mechanism of the considerable mechanical stiffening of DNA induced by the couple Zn2+/Calix[4]arene-1,3-O-diphosphorous acid
Source: Sci Rep. 2018 Jan 19;8:1226. doi: 10.1038/s41598-018-19712-4 (PMC5775194; doi:10.1038/s41598-018-19712-4)
Supplement: Supplementary file 1 — Supplementary Information [file 41598_2018_19712_MOESM1_ESM.pdf]

Elucidating the mechanism of the considerable mechanical stiffening of DNA induced by the couple Zn^{2+}/Calix[4]arene-1,3-O-diphosphorous acid

*Yannick Tauran[1,2] †, Mehmet C. Tarhan[2,3,4,5] †, Laurent Mollet[1] †, Jean Baptiste Gerves[1], Momoko Kumemura[2,3,4], Laurent Jalabert[2,4], Nicolas Lafitte[2,4], Ikjoo Byun[2,4], Beomjoon Kim[2,4], Hiroyuki Fujita[2,3,4], Dominique Collard[2,3,4], Florent Perret[6], Mickael Desbrosses[7], Didier Leonard[7], Christelle Goutaudier[1] and Anthony W. Coleman[1,2]**

[1]LMI CNRS UMR 5615, Université Lyon 1, Villeurbanne, 69622, France, anthony.coleman@univ-lyon1.fr
[2]LIMMS/CNRS-IIS UMI 2820, Institute of Industrial Science, The University of Tokyo, Tokyo, 153-8505, Japan.
[3]CNRS/IIS/COL/Lille 1 SMMiL-E project, 59046, Lille Cedex, France
[4]CIRMM, Institute of Industrial Science, The University of Tokyo, Tokyo, 153-8505, Japan.
[5]Univ. Lille, CNRS, Centrale Lille, ISEN, Univ. Valenciennes, UMR 8520 - IEMN, Lille, F59000, France
[6]Univ. Lyon, Université Claude Bernard Lyon 1, CNRS, CPE Lyon, ICBMS UMR 5246, 43 Boulevard du 11 Novembre 1918, F-69622 Lyon, France
[7]ISA, UMR 5280, Univ. Lyon 1, Villeurbanne F69100.

† Contributed equally to the work

* Anthony W. Coleman
LMI CNRS UMR5615, Université Lyon 1,
Villeurbanne, 69622, France
Tel: + 33 4 7243 1027
anthony.coleman@univ-lyon1.fr

Supplementary Figures

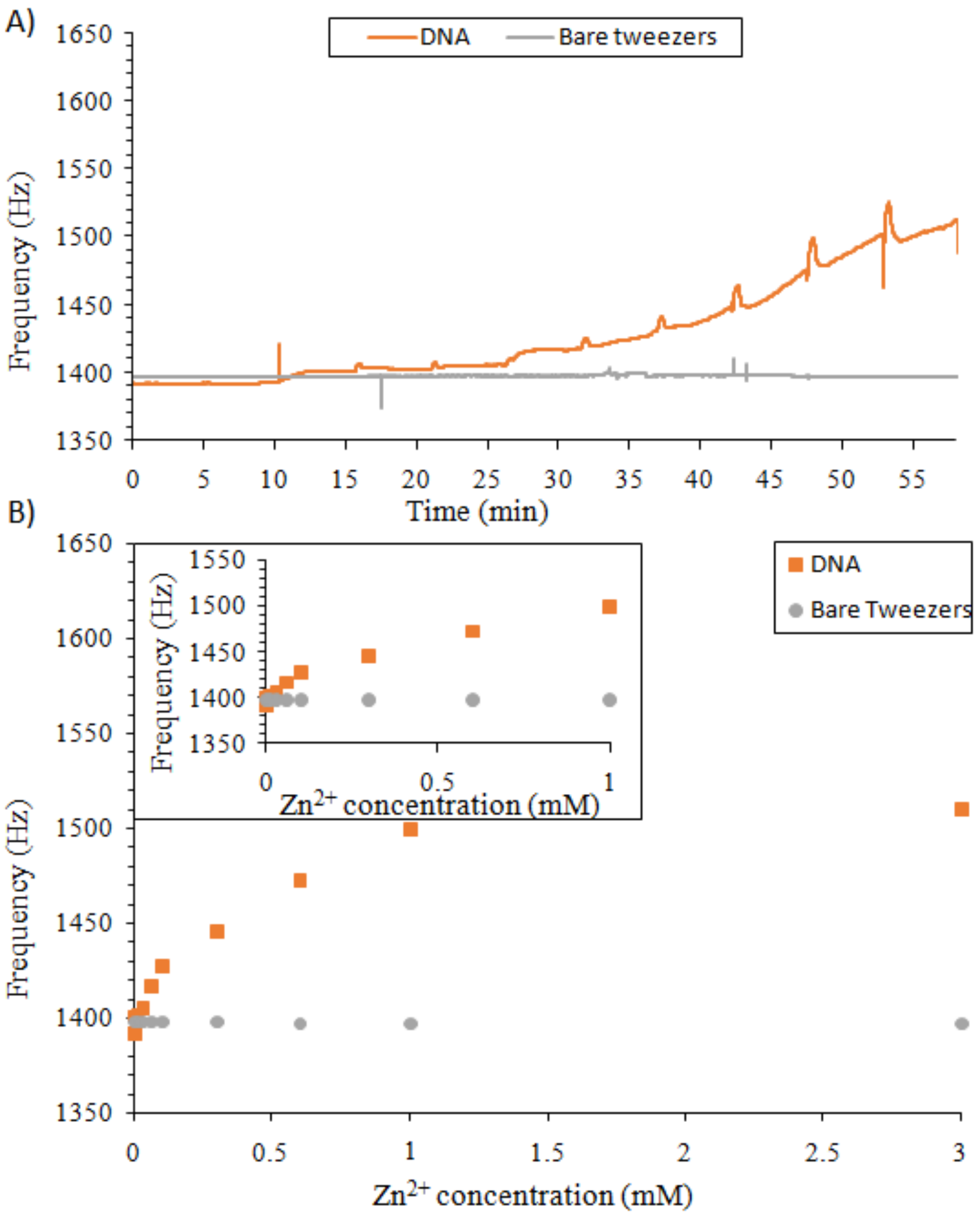

Supplementary Figure 1. A) DNA Resonance frequency after consecutive injections of Zn^{2+}concentration series (DI water, 0.0001 mM, 0.001 mM, 0.01 mM, 0.03 mM, 0.06 mM, 0.1 mM, 0.3 mM, 0.6 mM, 1 mM and 3 mM of Zn^{2+}) for 5 minutes and 20 seconds B) DNA Resonance frequency as a function of Zn^{2+} concentration. The inset corresponds to an enlargement at the lower Zn^{2+} concentrations.

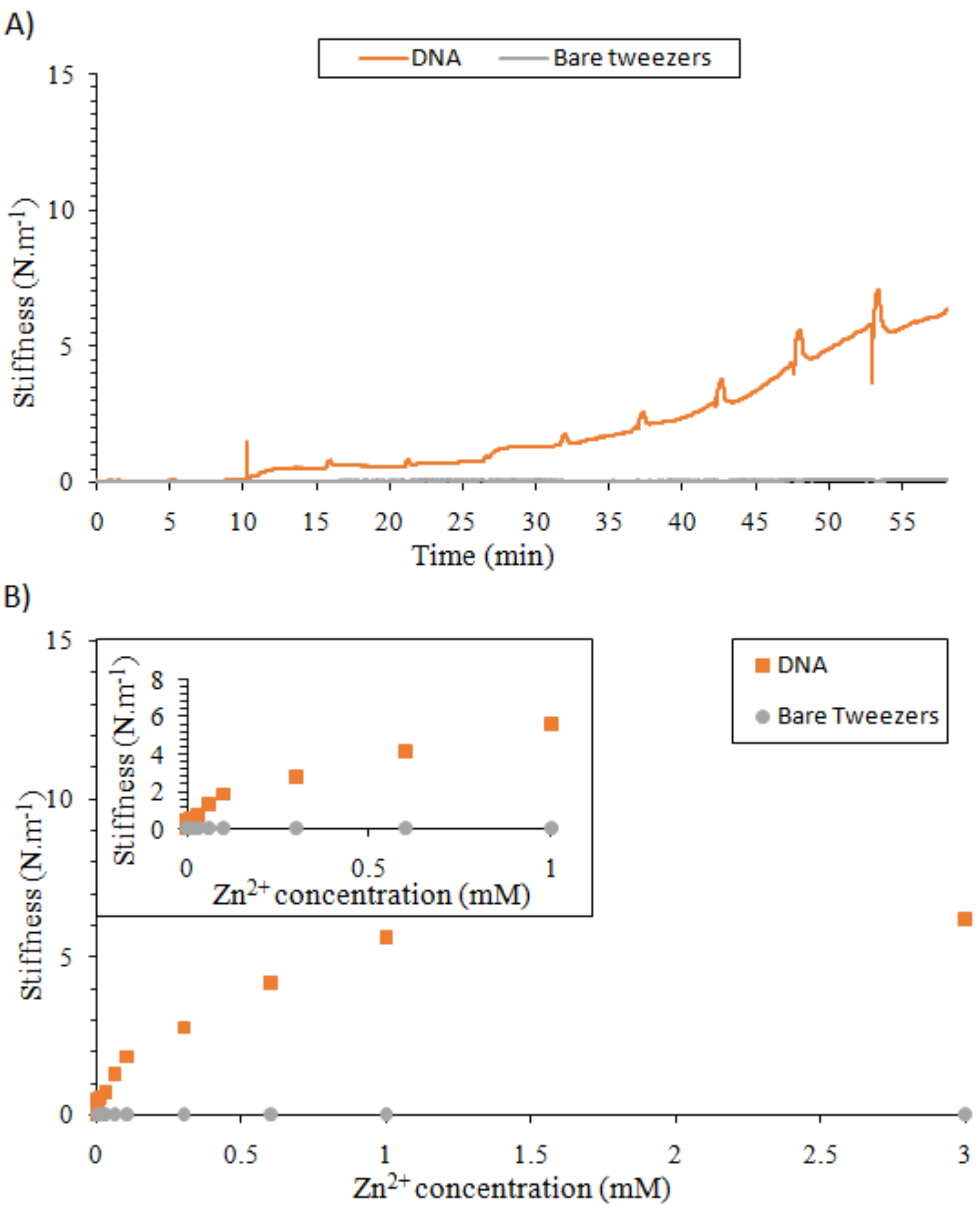

Supplementary Figure 2. A) DNA stiffness after consecutive injections of a Zn^{2+} concentration series (DI water, 0.0001 mM, 0.001 mM, 0.01 mM, 0.03 mM, 0.06 mM, 0.1 mM, 0.3 mM, 0.6 mM, 1 mM and 3 mM of Zn^{2+}) for 5 minutes and 20 seconds B) DNA stiffness as a matter of Zn^{2+} concentration. The inset corresponds to an enlargement at the lower Zn^{2+} concentrations.

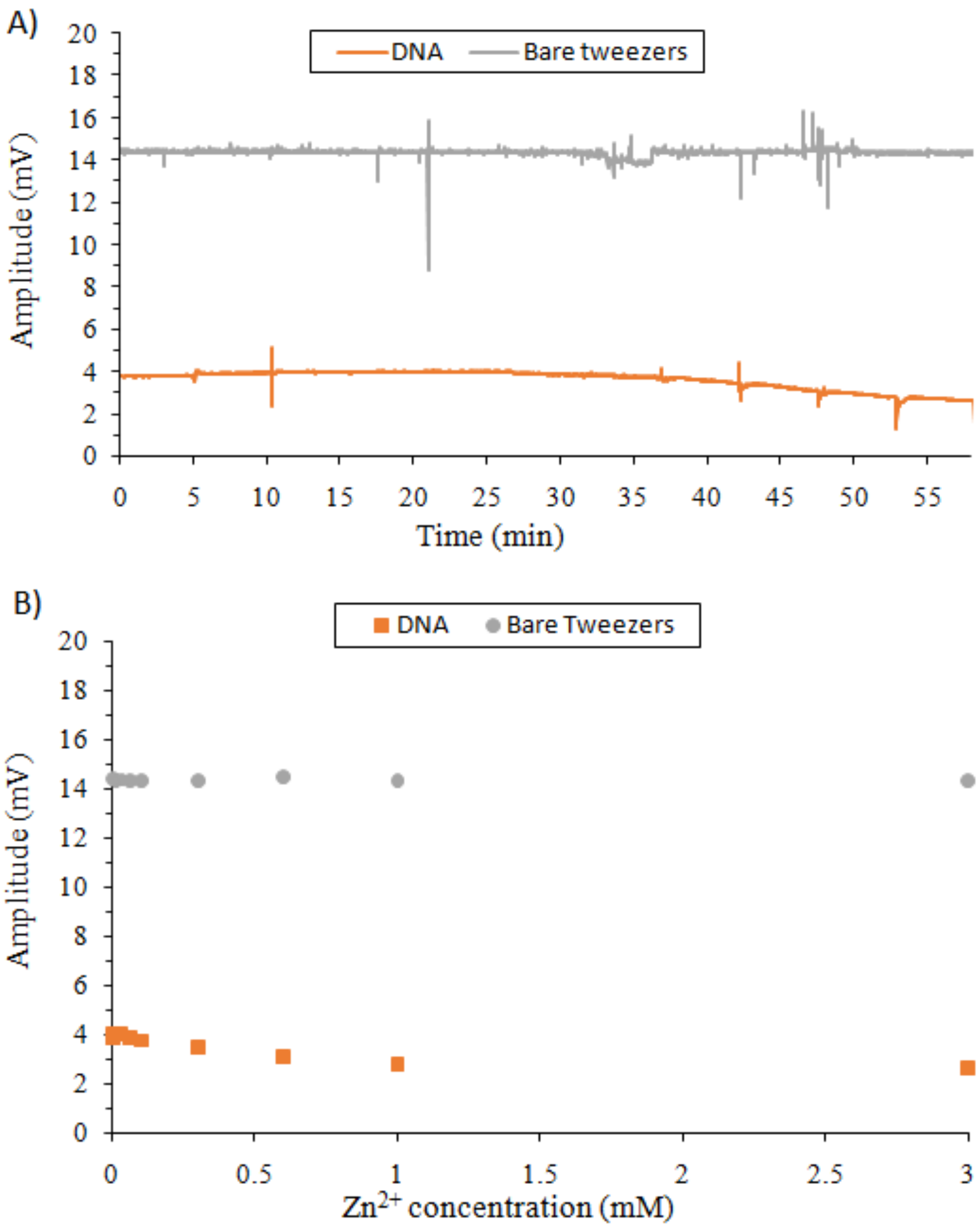

Supplementary Figure 3. A) DNA amplitude after consecutive injections of Zn^{2+} concentration series (DI water, 0.0001 mM, 0.001 mM, 0.01 mM, 0.03 mM, 0.06 mM, 0.1 mM, 0.3 mM, 0.6 mM, 1 mM and 3 mM of Zn^{2+}) for 5 minutes and 20 seconds B) DNA amplitude as a matter of Zn^{2+} concentration.

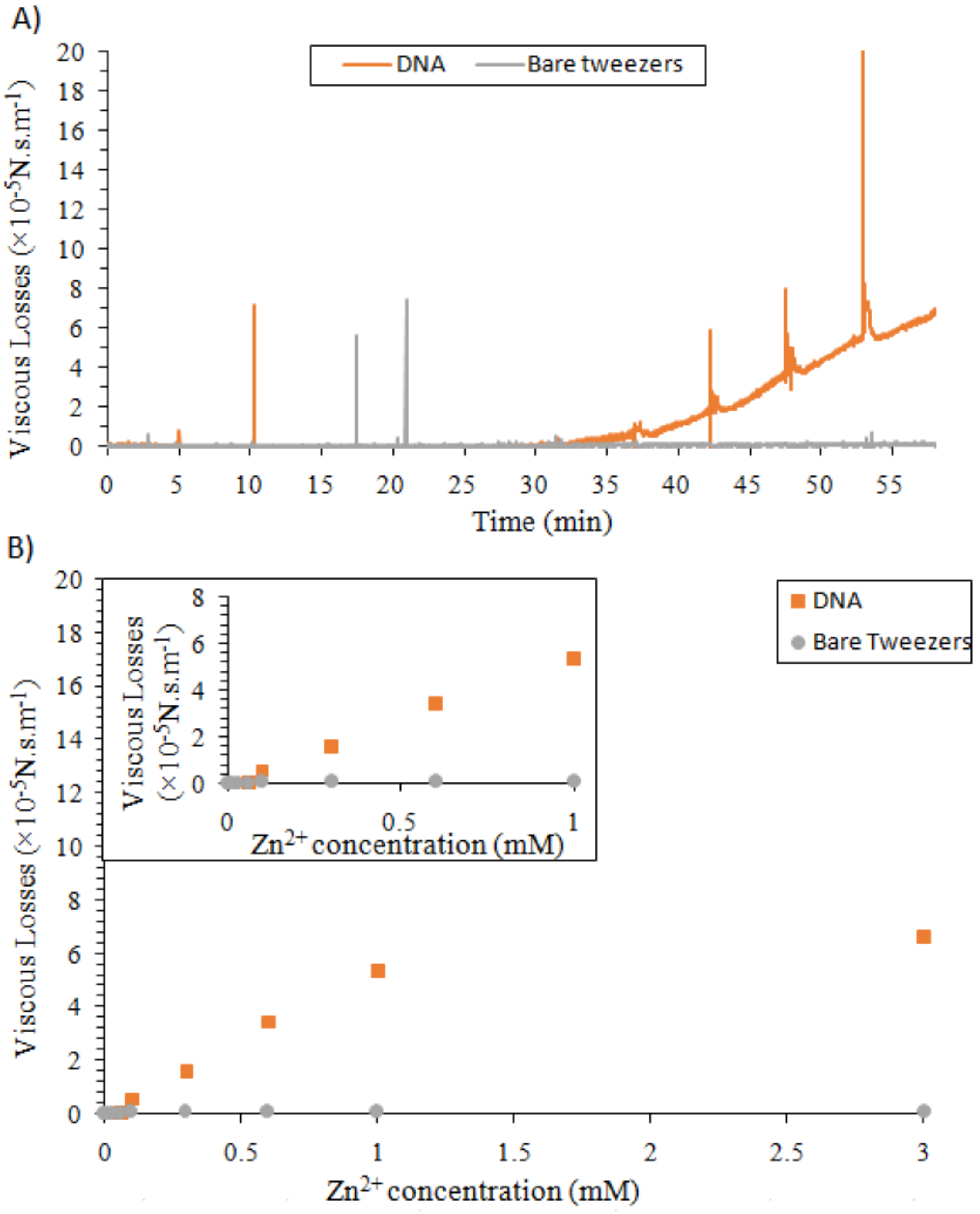

Supplementary Figure 4. A) DNA viscous losses after consecutive injections of Zn^{2+} concentration series (DI water, 0.0001 mM, 0.001 mM, 0.01 mM, 0.03 mM, 0.06 mM, 0.1 mM, 0.3 mM, 0.6 mM, 1 mM and 3 mM of Zn^{2+}) for 5 minutes and 20 seconds B) DNA viscous losses as a matter of Zn^{2+} concentration. The inset corresponds to an enlargement at the lower Zn^{2+} concentrations.

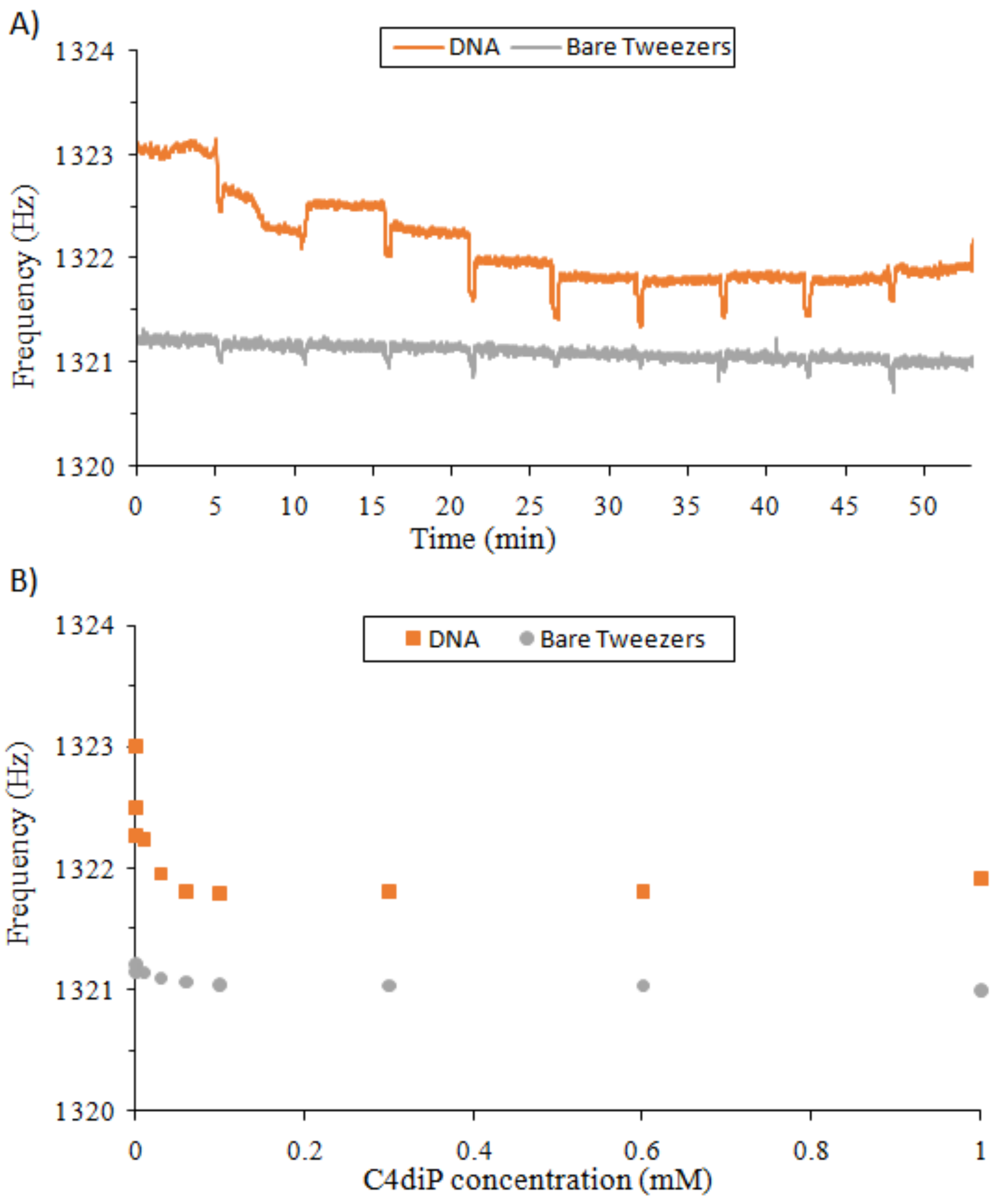

Supplementary Figure 5. A) DNA frequency after consecutive injections of C4diP concentration series (DI water, 0.0001 mM, 0.001 mM, 0.01 mM, 0.03 mM, 0.06 mM, 0.1 mM, 0.3 mM, 0.6 mM and 1 mM of C4diP) for 5 minutes and 20 seconds B) DNA frequency as a function of C4diP concentration.

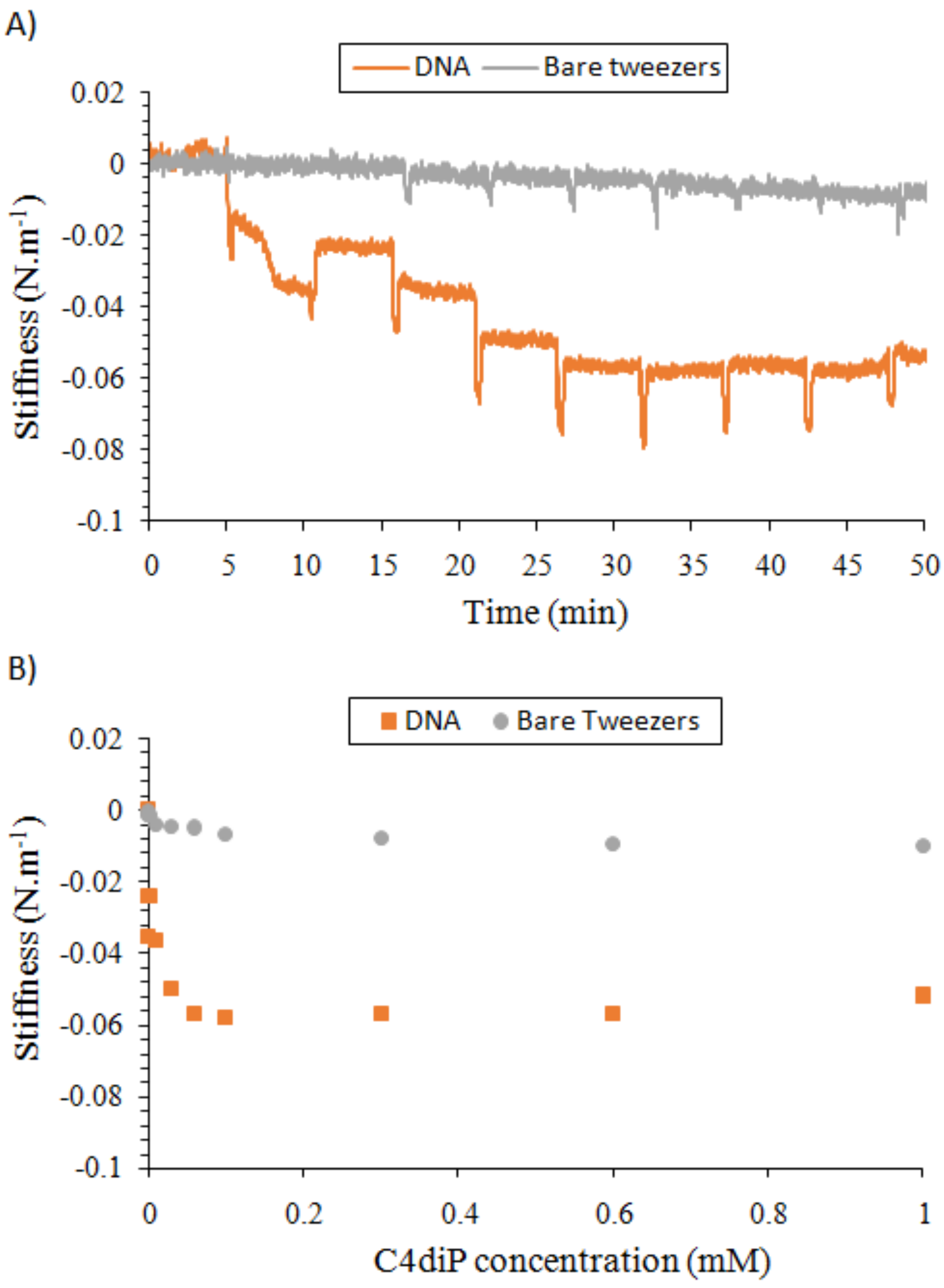

Supplementary Figure 6. A) Change of DNA stiffness after consecutive injections of C4diP concentration series (DI water, 0.0001 mM, 0.001 mM, 0.01 mM, 0.03 mM, 0.06 mM, 0.1 mM, 0.3 mM, 0.6 mM and 1 mM of C4diP) for 5 minutes and 20 seconds B) Change of DNA stiffness as a function of C4diP concentration.

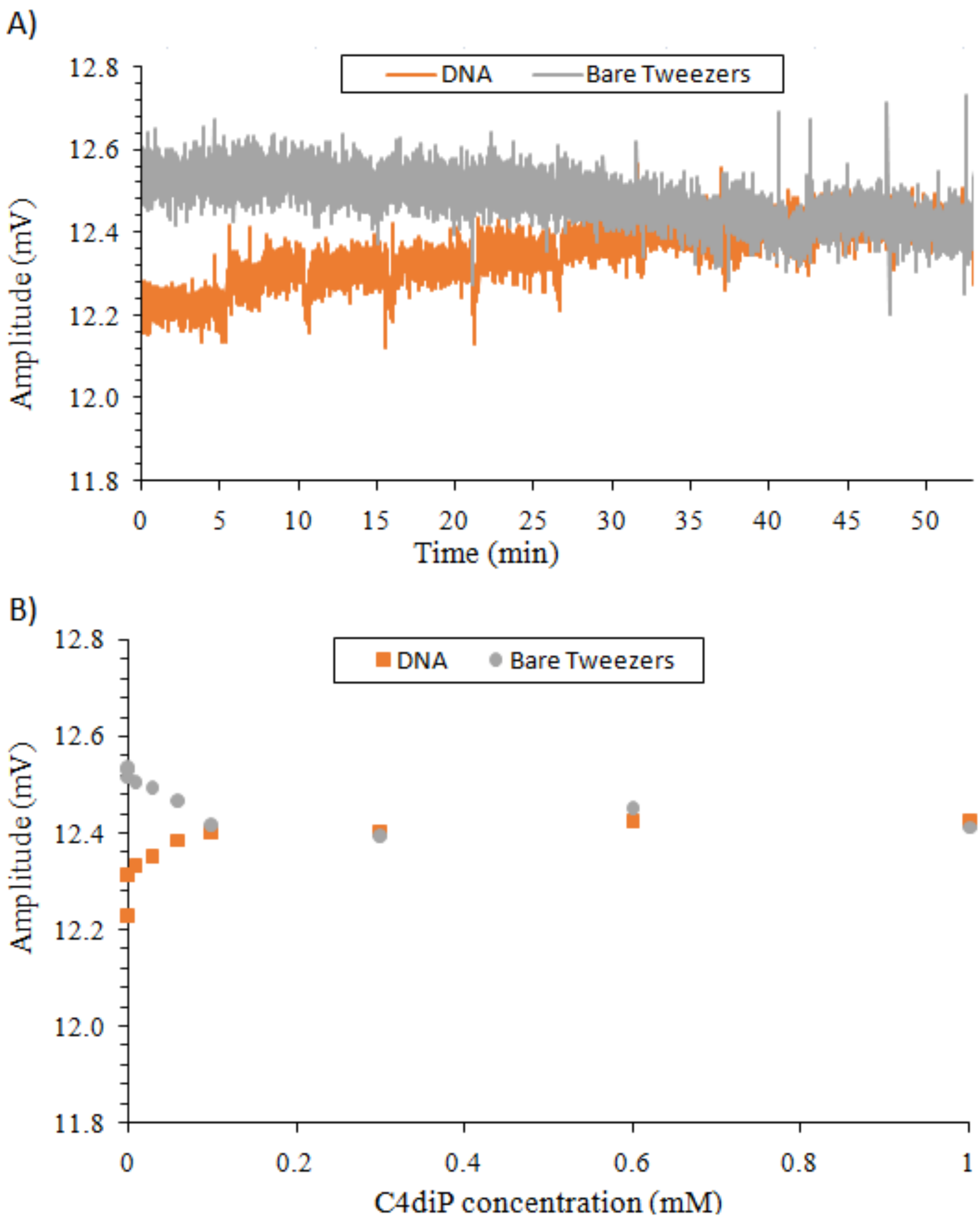

Supplementary Figure 7. A) DNA amplitude after consecutive injections of C4diP concentration series (DI water, 0.0001 mM, 0.001 mM, 0.01 mM, 0.03 mM, 0.06 mM, 0.1 mM, 0.3 mM, 0.6 mM and 1 mM of C4diP) for 5 minutes and 20 seconds B) DNA amplitude as a function of C4diP concentration.

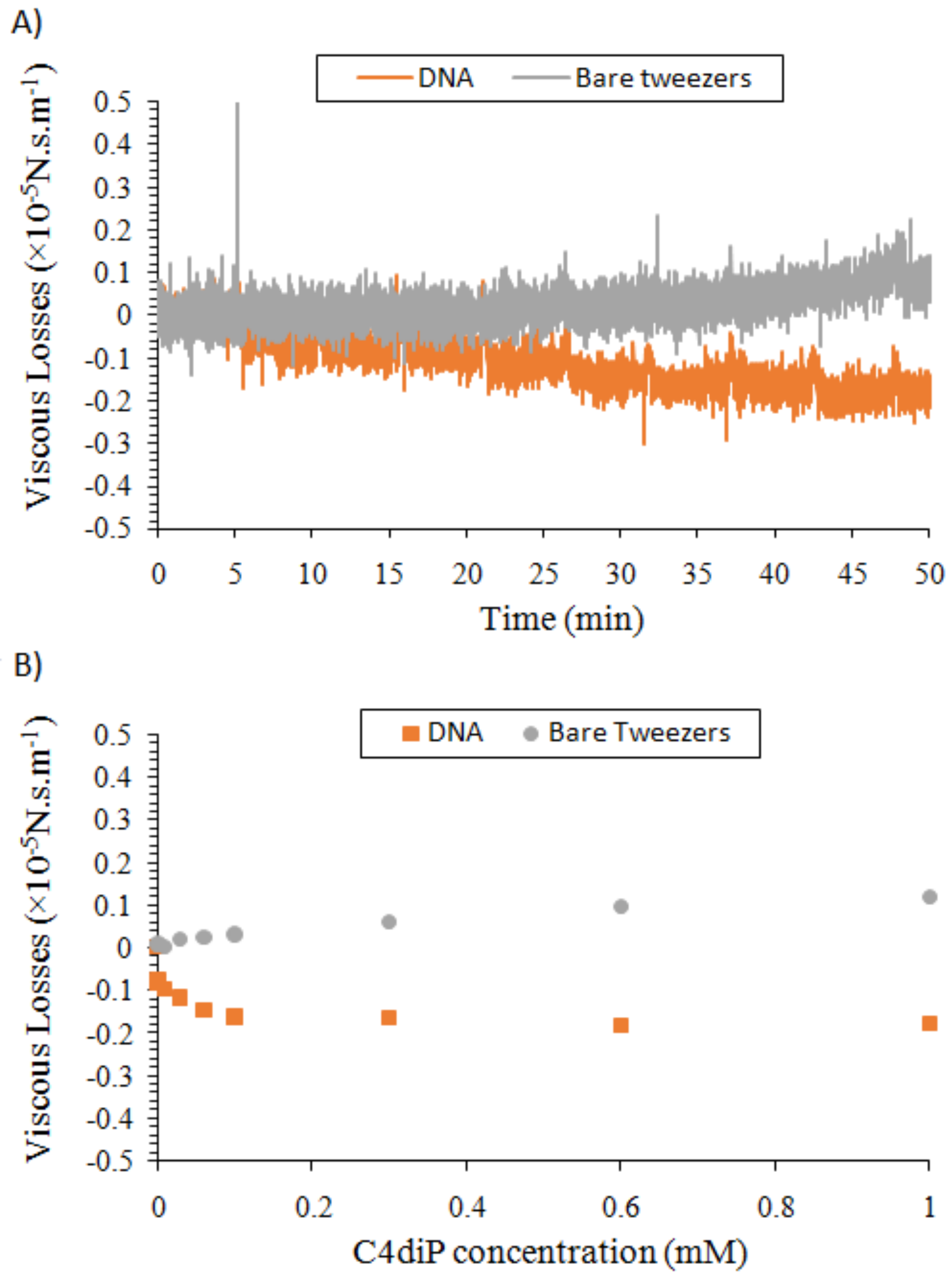

Supplementary Figure 8. A) Change of DNA viscous losses after consecutive injections of C4diP concentration series (DI water, 0.0001 mM, 0.001 mM, 0.01 mM, 0.03 mM, 0.06 mM, 0.1 mM, 0.3 mM, 0.6 mM and 1 mM of C4diP) for 5 minutes and 20 seconds B) Change of DNA viscous losses as a function of C4diP concentration.

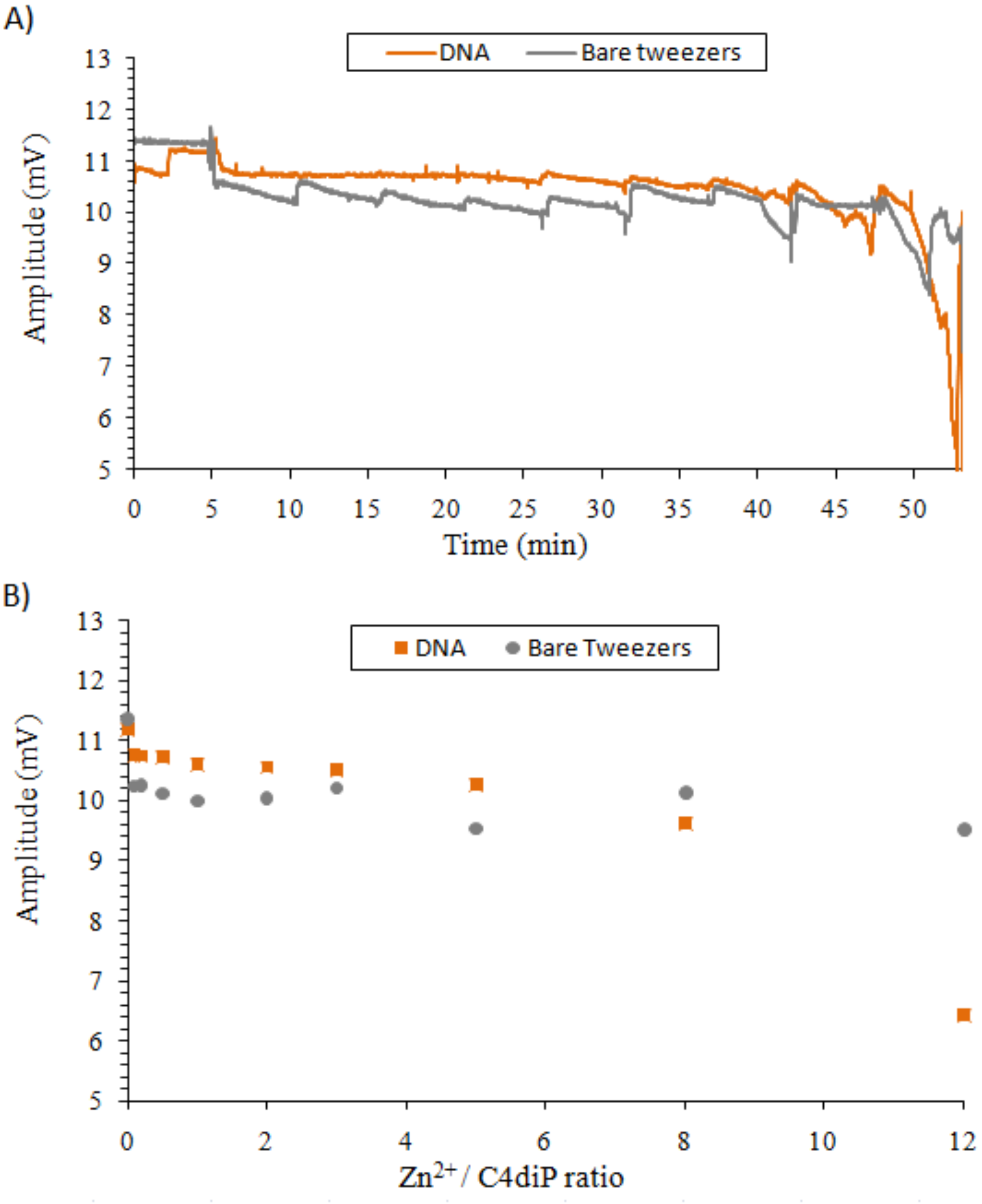

Supplementary Figure 9. A) DNA amplitude after consecutive injections of Zn^{2+}/C4diP ratio series (DI water, 0.1, 0.2, 0.5, 1, 2, 3, 5, 8 and 12) for 5 minutes and 20 seconds on a DNA bundle (orange curve) and on bare tweezers (grey curve) B) DNA amplitude as a function of Zn^{2+}/C4diP ratio on a DNA bundle (orange plots) and on bare tweezers (grey plots).

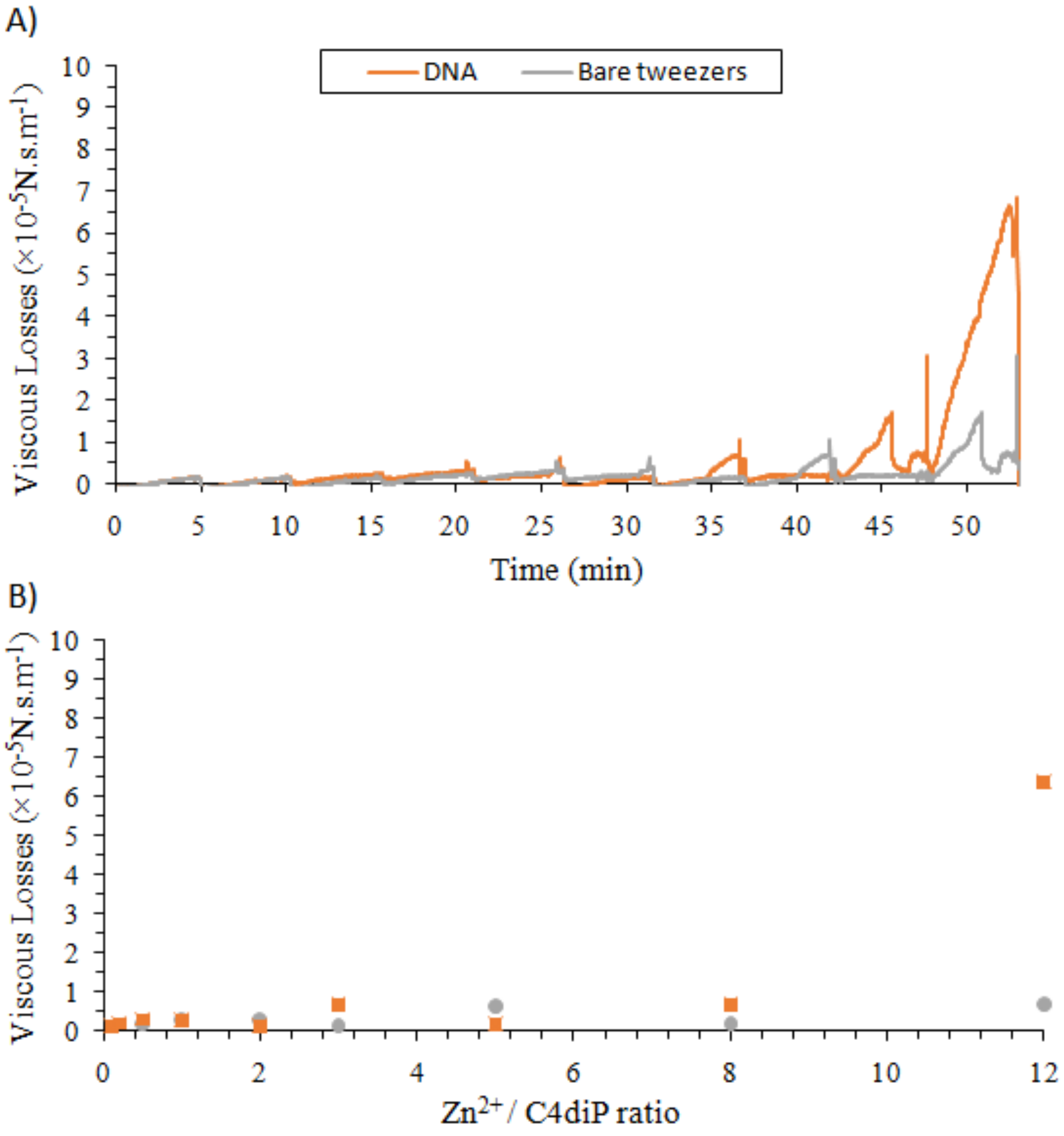

Supplementary Figure 10. A) DNA viscous losses after consecutive injections of Zn^{2+}/C4diP ratio series (DI water, 0.1, 0.2, 0.5, 1, 2, 3, 5, 8 and 12) for 300 seconds on a DNA bundle (orange curve) and on bare tweezers (grey curve) B) DNA viscous losses as a function of Zn^{2+}/C4diP ratio on a DNA bundle (orange plots) and on bare tweezers (grey plots).

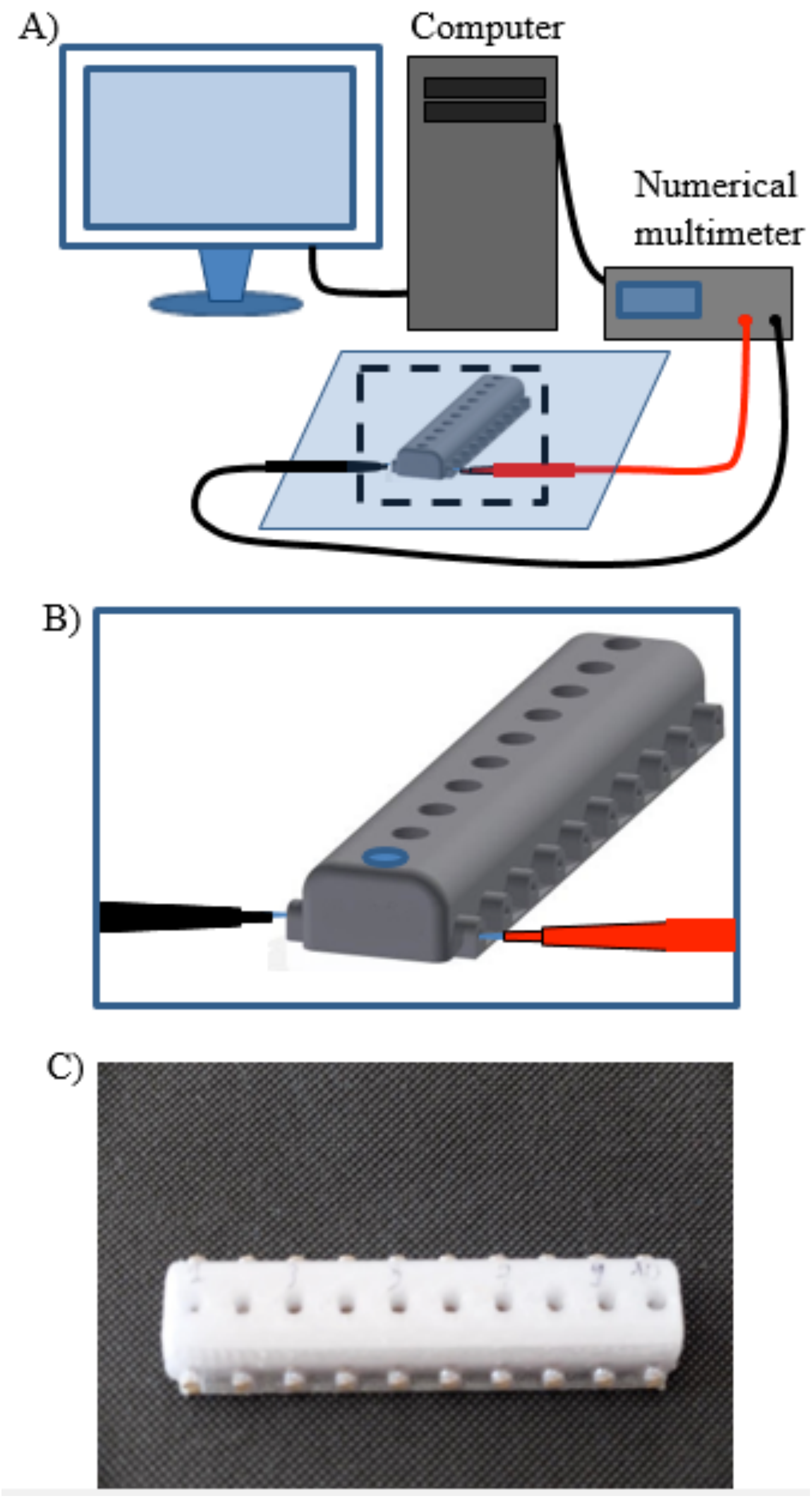

Supplementary Figure 11. Schematic representation of a set up for measuring solution/surface resistivity using a 3D printed device. In A) a numerical multimeter monitors the solution resistivity across the 3D device wells. The data are then collected and analysed on computer. In B) a magnified view of the 3D object containing 10 wells. The 3D device has

been designed using Inventor Professional software (Autodesk) C) Photograph of the electrochemical analytical device.

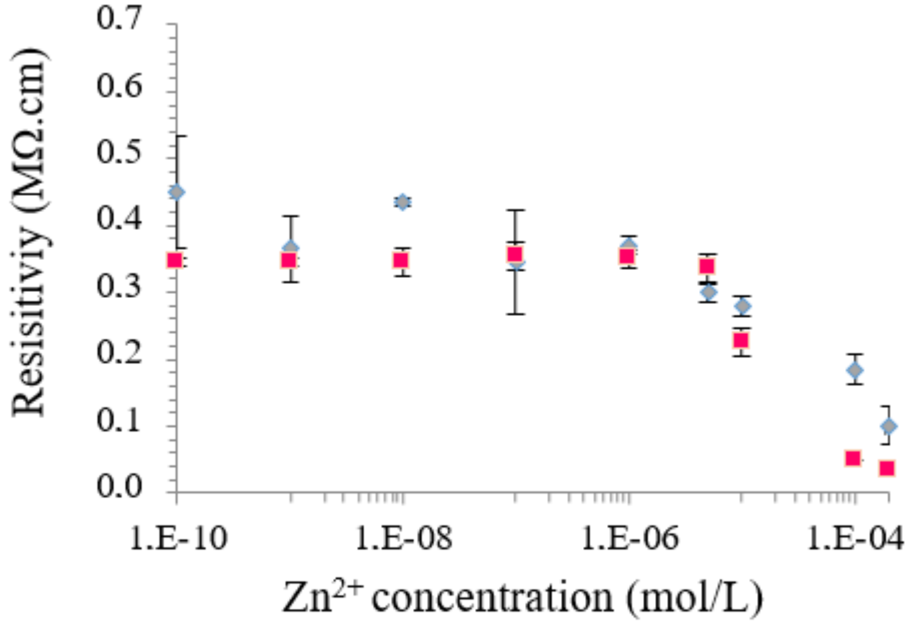

Supplementary Figure 12. Resistivity measurements of a concentration series of Zn^{2+} on AMP treated PET surfaces (red squares) and non-treated PET surfaces (blue diamonds).

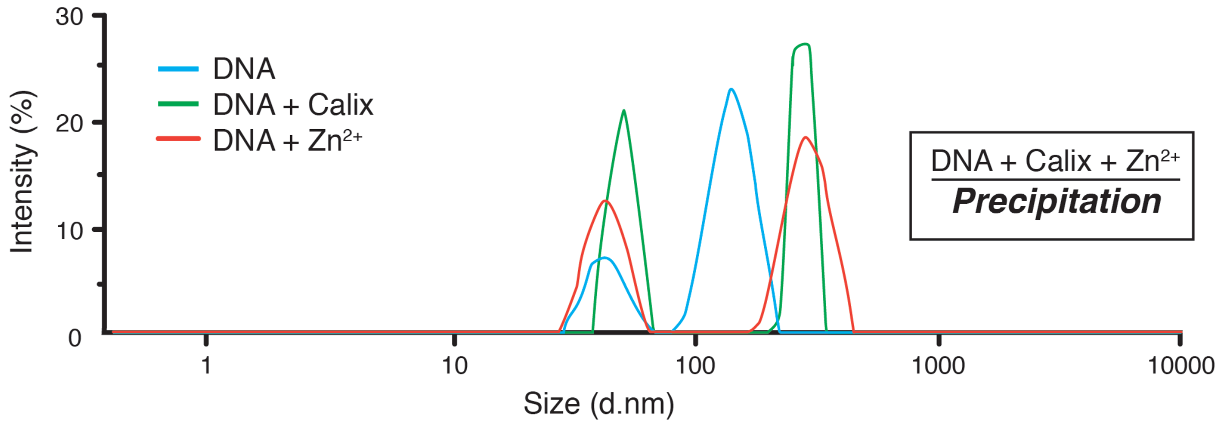

Supplementary Figure 13. Dynamic Light Scattering experiments undertaken for DNA only (blue), DNA in the presence of C4diP (green), DNA in the presence of Zn^{2+} (red), and DNA in the presence of C4diP and Zn^{2+} at a ration of 1:10 when no objects can be observed due aggregation. All measurements were taken 30 minutes after mixing.
